# DIGGER 2.0: digging into the functional impact of differential splicing on human and mouse disorders

**DOI:** 10.1093/nar/gkaf384

**Published:** 2025-05-08

**Authors:** Elias Albrecht, Konstantin Pelz, Alexander Gress, Hieu Nguyen Trung, Olga V Kalinina, Tim Kacprowski, Jan Baumbach, Markus List, Olga Tsoy

**Affiliations:** Data Science in Systems Biology, TUM School of Life Sciences, Technical University of Munich, Maximus-von-Imhof Forum 3, 85354 Freising, Germany; Institute for Computational Systems Biology, University of Hamburg, Albert-Einstein-Ring 8-10, 22761 Hamburg, Germany; Data Science in Systems Biology, TUM School of Life Sciences, Technical University of Munich, Maximus-von-Imhof Forum 3, 85354 Freising, Germany; Helmholtz Institute for Pharmaceutical Research Saarland (HIPS), Helmholtz Centre for Infection Research (HZI), Campus E8.1, 66123 Saarbrücken, Germany; Graduate School of Computer Science, Saarland University, Campus E1.3, 66123 Saarbrücken, Germany; Data Science in Systems Biology, TUM School of Life Sciences, Technical University of Munich, Maximus-von-Imhof Forum 3, 85354 Freising, Germany; Helmholtz Institute for Pharmaceutical Research Saarland (HIPS), Helmholtz Centre for Infection Research (HZI), Campus E8.1, 66123 Saarbrücken, Germany; Drug Bioinformatics, Medical Faculty, Saarland University, Gebäude 15, 66421 Homburg, Germany; Center for Bioinformatics, Saarland University, Campus E2.1, 66123 Saarbrücken, Germany; Division Data Science in Biomedicine, Peter L. Reichertz Institute for Medical Informatics of Technische Universität Braunschweig and Hannover Medical School, Rebenring 56 Lower Saxony, 38106 Braunschweig, Germany; Braunschweig Integrated Centre of Systems Biology (BRICS), Technische Universität Braunschweig, Rebenring 56 Lower Saxony, 38106 Braunschweig, Germany; Institute for Computational Systems Biology, University of Hamburg, Albert-Einstein-Ring 8-10, 22761 Hamburg, Germany; Institute of Mathematics and Computer Science, University of Southern Denmark, Campusvej 55, 5230 Odense, Denmark; Data Science in Systems Biology, TUM School of Life Sciences, Technical University of Munich, Maximus-von-Imhof Forum 3, 85354 Freising, Germany; Munich Data Science Institute (MDSI), Technical University of Munich, Walther-von-Dyck-Straße 10, 85748 Garching, Germany; Institute for Computational Systems Biology, University of Hamburg, Albert-Einstein-Ring 8-10, 22761 Hamburg, Germany

## Abstract

Changes in alternative splicing between groups or conditions contribute to protein–protein interaction rewiring, a consequence often neglected in data analysis. The web server and database DIGGER overcomes this limitation by augmenting a protein–protein interaction network with domain–domain interactions and splicing information. Here, we present DIGGER 2.0, which now features both experimental and newly added predicted domain–domain interactions. In addition to the human interactome, DIGGER 2.0 adds support for mouse as an important model organism. Additionally, we integrated the splicing analysis tool NEASE, which allows users to perform online splicing- and interactome-informed enrichment analysis on RNA-seq data. In two application cases (multiple sclerosis and mice models of cardiac diseases), we show the utility of DIGGER 2.0 for deeper exploration and functional interpretation of changes in alternative splicing in human and mouse disorders. DIGGER 2.0 is available at https://exbio.wzw.tum.de/digger/.

## Graphical Abstract

**Graphical Abstract:**
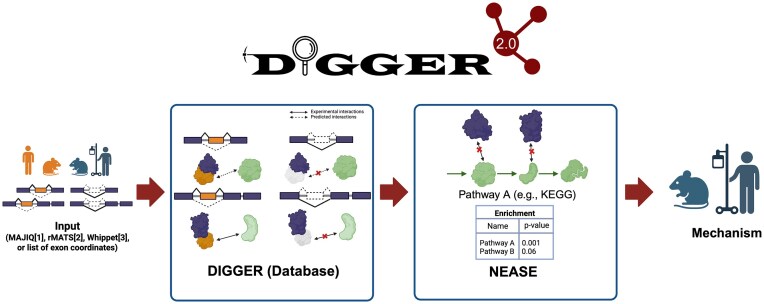

## Introduction

Alternative splicing (AS) allows one gene to encode for multiple proteins (proteoforms or isoforms) [1]. These protein isoforms can differ in sequence and structure and may comprise different domains. The protein sequence, and specifically the resulting domain composition of a protein, determines its interaction partners. Indeed, isoforms of the same protein may share less than half of their interaction partners [2], and hence, protein–protein interaction (PPI) networks should be resolved at the isoform level. However, tools offering both a large-scale analysis of the consequences of AS on protein interactome and functional interpretation are not abundant and often lack core functionality (Table 1).

**Table 1.** Tools that analyze functional consequences of AS on protein structure

Tool	Source code available	Data downloadable	Input	Uses DDIs	Large-scale data analysis	Functional interpretation	General inputs	Tissue-specific analysis	Pathway analysis	Comments
ALT-IN [8]	$\checkmark$	$\checkmark$	PPIs, isoform sequence		$\checkmark$					
CoSpliceNet [9]			RNA-seq			$\checkmark$			$\checkmark$	Plants only
DeepIII [10]	$\checkmark$	$\checkmark$	RNA-seq, exon array, DDIs, isoform sequence	$\checkmark$	$\checkmark$					
DoChaP [11]	$\checkmark$		Gene ID			$\checkmark$	$\checkmark$			Unavailable
DomainGraph [12]			AltAnalyze [12] output			$\checkmark$	$\checkmark$		$\checkmark$	Unavailable
ExonSkipDB [13]		$\checkmark$	Gene ID		n/a	$\checkmark$		$\checkmark$		
Splitpea [14]	$\checkmark$		DDIs, differential exon usage	$\checkmark$				$\checkmark$		
tappAS [15]	$\checkmark$	$\checkmark$	RNA-Seq, gene annotations		$\checkmark$	$\checkmark$	$\checkmark$			
LINDA [16]	$\checkmark$	$\checkmark$	TF activity, exon skipping events	$\checkmark$		$\checkmark$	$\checkmark$		$\checkmark$	
DIGGER [4]	$\checkmark$	$\checkmark$	Gene, ID transcript ID, protein ID		n/a		$\checkmark$			
NEASE [3]	$\checkmark$	$\checkmark$	Exon skipping events	$\checkmark$		$\checkmark$	$\checkmark$		$\checkmark$	
DIGGER 2.0	$\checkmark$	$\checkmark$	Exon skipping events, gene ID, transcript, protein ID	$\checkmark$	$\checkmark$	$\checkmark$	$\checkmark$		$\checkmark$	

To fill this gap, we previously developed NEASE [3] and DIGGER 1.0 [4]. DIGGER is a database that constructs and stores a PPI network augmented by domain–domain interactions (DDIs), interactions with residue-level evidence, and domain–motif interactions (DMIs). DIGGER offers a detailed view of how an AS event rewires these interactions; however, it does not reveal molecular functions affected by these splicing changes. We addressed this limitation in a separate tool, NEASE, available as a Python package. NEASE identifies biochemical pathways and genes that rewire PPIs with these pathways due to changes in AS between groups or conditions. It then employs an edge-level hypergeometric test to detect the pathways where the number of rewired interactions is significantly higher than expected by chance. NEASE uses the results from AS detection tools, e.g. rMATS, as input and reports affected pathways.

In this manuscript, we present DIGGER 2.0, a web tool that combines and broadens the functionality of DIGGER 1.0 and NEASE. DIGGER 2.0 enables fast and easy online functional enrichment analyses of changes in AS by integrating the NEASE method into the website. Beyond the existing support for the human interactome, DIGGER 2.0 adds support for mouse as an important model organism. In addition, DIGGER 2.0 also increases the number of PPIs augmented by DDIs integrating high-confidence DDI predictions based on the PPI domain miner (PPIDM) method [5].

We demonstrate the utility of DIGGER 2.0 in two scenarios: First, we re-evaluate a use case of multiple sclerosis (MS) RNA-seq data analysis reported in the original NEASE manuscript [3] and highlight the added value of the predicted DDIs (pDDIs). Second, we analyzed two RNA-seq datasets from mice models of cardiac diseases [6, 7] and found potential candidates involved in disease development that could not be detected by the gene-level analysis.

Overall, DIGGER 2.0 enables deeper functional interpretation of differential splicing in human and mouse data with just a few clicks.

## Materials and methods

### Construction of the mouse interactome

We constructed the joint PPI/DDI mouse interactome following the same approach as for the human interactome described in the original DIGGER publication [4]. We retrieved PPIs from BioGRID version Mus_musculus-4.4 [17] and downloaded experimentally validated DDIs from 3did (v2020_5) [18] and DOMINE (2011) [19]. Next, we used the BioMART mapping tool (Ensembl 109) [20] to map exons and protein domains via transcript IDs by checking for overlaps in their respective genomic coordinates.

For the integration of NEASE, we collected and extracted additional data: DMIs from the Eukaryotic Linear Motif server [21], interactions with residue-level evidence based on the co-resolved structures in the Protein Data Bank (PDB) [22], and metabolic pathways from the ConsensusPathDB [23].

### DDI prediction

We predicted DDIs using the PPI domain miner (PPIDM) [5]. PPIDM uses the observation that the same domain type might be used in different proteins. The method connects domains with the proteins they occur in and calculates how often the same pair of domain types co-occur in different proteins participating in PPIs across several databases. The more often they appear together in PPIs and the more often this PPI is supported across multiple resources, the higher the resulting score. Additionally, the contribution of each resource is weighted based on the resource reliability. PPIDM evaluates the resulting scores using the ground truth DDIs from 3did [18] and KBDOCK [24] databases and defines three levels of confidence: gold, silver, and bronze.

For the human joint PPI/DDI network, we retained the original PPIDM weights but recalculated the confidence level threshold based on the ground-truth DDIs from the 3did as the KBDOCK data is no longer available. Our recalculated threshold remained very close to the original publication (0.015 versus 0.01586).

To predict DDIs for the mouse joint PPI/DDI network, we generalized PPIDM as it was originally designed for human data. We calculated the scores based on PPIs from six different databases: BioGRID version 4.4.230 [17], the Mippie database version v1.0 [25], both SPRING exp and rest DB version v12.0 [26], Intact (downloaded on 16 February 2024) [27], MINT (downloaded on 4 March 2024) [28], and DIP version 20170205 [29]. We analyzed 10 000 random parameter combinations with parameters ranging from 1 to 100 in integer steps. They correspond to the weighted contribution of each resource that supports PPI to the final score. We then compared the top-scored pDDIs with the ground truth DDIs (see Supplementary Fig. S2 and Supplementary File for more details). Since the information about mouse PPIs is sparse compared to the information about human PPIs, the initial scores were low. We boosted the PPIDM scores by adding an additional database with homology-based PPIs as outlined below.

Specifically, we applied StructMAn [30] to map the mouse proteome to three-dimensional protein structures from the PDB [22], including homologs of the target mouse proteins. Whenever two different target proteins are mapped to an experimentally resolved protein complex in PDB, StructMAn calculates all interaction interfaces between all proteins in this complex and checks whether the two mapped proteins also interact with each other there. Identification of an experimentally resolved interaction between homologs is a strong indicator to infer a PPI between target mouse proteins. By aligning the mouse proteins to the corresponding chains in this complex, StructMAn identifies individual amino acids that participate in an interaction interface. This information can be used to check whether alternatively spliced domains are participating in the identified interactions. A similar protein structure-based method of PPI/DDI inference was already implemented in DIGGER 1.0, but without accounting for homologs. Here we expand the human interactome, too, taking the structures of homologous proteins into account and infer over 11 million PPIs that are supported by structural evidence. These PPIs are used as supporting evidence for PPIDM in the prediction for mouse.

### NEASE integration

We integrated the original NEASE Python package into the backend framework of DIGGER 2.0 with two main additions: the mouse interactome and pDDIs for both human and mouse networks. We also changed how the method computes the PPI network characteristics. While the original NEASE uses precomputed values to assess the network size and the number of interactions with pathways, this is not feasible for all combinations of pDDI confidence levels. Therefore, we now compute the network characteristics on the fly.

### The impact of pDDIs on AS data analysis: the MS use case

To evaluate the impact of pDDIs on AS analysis, we re-evaluated the analysis of the MS dataset (GSE138614) initially performed in [31]. We used the results of the MAJIQ [32] differential splicing analysis of normal-appearing white matter and active lesion regions from postmortem white matter brains of MS patients from the original NEASE publication. Next, we performed the NEASE analysis with and without high and medium confidence level pDDIs while leaving the other parameters at their default values.

### Mouse data analysis

We used the raw RNA-seq data of the cardiac disease mouse models from [6] and [7] (GSE275835 and GSE182985, respectively). From [6], we compared the animals with the ischemic reperfusion injury with controls. From [7], we chose the 8W-post-TAC samples as we expected a stronger signal in mice exposed for a longer time to pressure overload. For the differential splicing analysis, we used Whippet [33] and rMATS [34] with the reference genome annotation mus_musculus_GRCm39_112, and used the default parameters. The results were analyzed with NEASE with and without high confidence level pDDIs. We also applied different thresholds to define differentially used AS events for deltaPSI [the difference between percentage spliced in (PSI) values in different conditions] and *P*-value. For differential gene expression analysis of the data from [6], we used the raw counts published at GSE275835 and the edgeR online implementation in the Degust package [35]. For [7], we first mapped the raw RNA-seq reads on the *Mus musculus* genome with STAR 2.7.5 [36] and obtained the raw counts with featureCounts (part of the Subread 2.0.0) [37]. Next, we again used the edgeR implementation in Degust [35]. For both datasets, we used the thresholds of |logFC| > 1 and *P*-adjusted ≤.05.

## Results

### Construction of the mouse interactome

DIGGER 2.0 supports not only human but also mouse molecular interaction networks. The networks are stored as joint PPI/DDI networks where PPI edges from BioGRID are additionally augmented by DDIs, DMIs, and interactions with residue-level evidence [3]. We constructed a PPI/DDI joint network for mouse based on the BioGRID PPIs. PPIs were augmented based on 8455 Pfam domains with 13 348 DDIs, 230 DMIs, and 559 residue-level interactions. Our final joint PPI/DDI mouse molecular interaction network consists of 4936 proteins and 13 476 interactions.

### Integration of pDDIs

We augmented more PPI edges from BioGRID using DDI predictions for both human and mouse. Many methods for DDI predictions suffer from data leakage, do not provide an interpretable output, or do not provide the source code [5, 8, 10, 38, 39]. We thus chose to expand DIGGER using PPIDM [5], as it relies on a simple statistical approach, offers an interpretable output, and provides source code that allowed us to reproduce the results from the original publication.

First, we applied the PPIDM method to predict DDIs for the human interactome. DIGGER 1.0 contained 9996 proteins from BioGRID [17] with 52 467 PPIs augmented by DDIs from DOMINE and 3did [18, 19], and 7116 PPIs augmented by DMIs and interactions with residue-level evidence. With pDDIs, we additionally augmented 7232 PPIs, corresponding to a 14% increase in annotated PPIs.

For the pDDIs in the mouse interactome, we expanded the number of augmented PPIs from 13 348 to 15 634, corresponding to a 17% increase in annotated mouse PPI interactions.

### NEASE analysis

**Figure 1.**
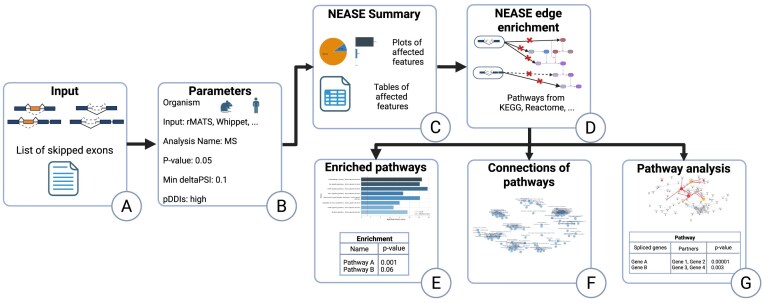
Overview of the NEASE workflow. (**A**) The input data include differentially used exon skipping events as a list or output from MAJIQ, rMATS, or Whippet. (**B**) The user selects parameters of the NEASE analysis. (**C**) DIGGER 2.0 performs the analysis of affected protein features. (**D**) DIGGER 2.0 performs the splicing-aware pathway enrichment based on the chosen database (e.g. KEGG, Reactome). (**E**) DIGGER 2.0 results include the list of enriched pathways. (**F**) KEGG and Reactome pathways can be visualized as clusters. (**G**) The user can analyze an individual pathway.

DIGGER 2.0 offers to perform online splicing-aware network enrichment with NEASE. Figure 1 shows the main steps of the NEASE data analysis.

Users start by uploading a list of exon skipping events: a list of exon coordinates or outputs from differential splicing analysis tools such as rMATS, MAJIQ, or Whippet (Fig. 1A). Once the data have been uploaded, users can choose the organism (human or mouse), the thresholds for filtering out significantly changed events (*P*-value and deltaPSI), and whether they want to use pDDIs (Fig. 1B).

DIGGER 2.0 outputs the list of proteins with domains affected by changes in AS (Fig. 1C). Next, users can choose the reference database (e.g. KEGG [40], Reactome [41], and WikiPathways [42]) and run splicing-aware network enrichment analysis (Fig. 1D). At this stage, DIGGER 2.0 runs the edge-level hypergeometric test to detect with which pathways AS changes rewired interactions more often than by chance (Fig. 1E). For Reactome and KEGG, DIGGER 2.0 also visualizes the connections between enriched pathways (Fig. 1F).

Next, users can investigate the impact of changes in AS on an individual pathway (Fig. 1G). DIGGER 2.0 outputs and visualizes the interactions between proteins from the pathway of interest, as well as proteins that rewire interactions with this pathway due to changes in AS. The latter might either belong or not belong to the pathway of interest.

Finally, all the results can be downloaded or shared via a link that is preserved for up to 6 months.

### The case study of MS

MS is a chronic autoimmune disease resulting in damage to cells in the central nervous system. The symptoms include fatigue and visual, motor, and sensory problems [43]. The etiology of MS is unclear [44].

The analysis in the NEASE original publication [3] detected 24 affected protein domains, 1 DMI, and 7 interactions with residue-level evidence. In total, 182 protein interactions were affected by changes in AS. We repeated the NEASE analysis, adding high and medium confidence level pDDIs. The number of affected domains, DMIs, and interactions with residue-level evidence remained the same. However, we detected 341 affected interactions.

**Figure 2.**
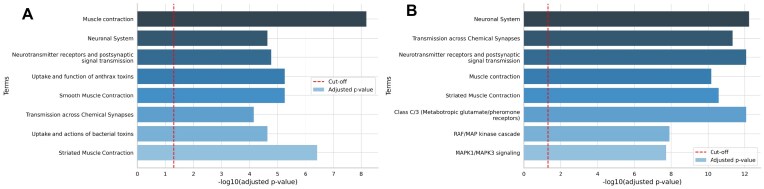
Most significant enrichment results sorted by NEASE score; (**A**) without predicted interactions; (**B**) using high- and medium-confidence predicted interactions. Overall, both methods have a high agreement, but some pathways have gained more importance (lower *P*-values) when using pDDIs.

Among the enriched Reactome pathways, 10 of the top 15 pathways remained identical in both analyses, including those related to the neuronal system and muscle contraction (Fig. 2). Notably, the addition of pDDIs results in lower *P*-values in the hypergeometric enrichment test. Similarly, pathways related to MAPK signaling cascades (“MAPK family signaling cascades,” “MAPK1/MAPK3 signaling,” and “RAF/MAP kinase cascade”) also have lower *P*-values in the re-analysis (e.g. 0.0024 versus 0.00000024 for “MAPK family signaling cascades”), and MAPK kinases are known to be activated in patients with MS and are potential therapeutic targets [45]. This observation might indicate that changes in AS have a more prominent impact on all these processes.

Indeed, in the re-analysis, we detected more genes that might lose interaction partners in the MS-related pathways due to changes in AS. For example, we found two more genes that rewire interactions with the “Muscle contraction” pathway: DMD (dystrophin) and SYNE1 (spectrin repeat containing nuclear envelope protein 1). DMD is involved in the cytoskeletal structure formation [46]. Changes in AS of DMD might rewire interactions with itself, TNNC1 (troponin C1), and TNNC2 (troponin C2). TNNC1 and TNNC2 encode the troponin C components from the cardiac and skeletal muscle, respectively, and are key players in regulating striated muscle contraction [47, 48]. SYNE1 does not belong to the “Muscle contraction” pathway; however, changes in AS might affect interactions with DMD and TNNC1.

Similarly, we found one more gene that loses interactions with the “Neuronal system” pathway: ALDH3A2 (aldehyde dehydrogenase isozyme) that is responsible for detoxifying aldehydes. There is evidence that reactive aldehydes contribute to the pathogenesis of MS [49].

In the re-analysis with pDDIs, we also find the glutamate-related pathways [“Class C/3 (Metabotropic glutamate/pheromone receptors),” “Glutamate binding, activation of AMPA receptors and synaptic plasticity,” and “Unblocking of NMDA receptor, glutamate binding and activation”]. Glutamate and its receptors are essential in the neuronal communication network. Excessive stimulation by glutamate was shown to damage or even kill neurons, an effect called glutamate excitotoxicity [50–52].

Thus, the addition of pDDIs reveals more mechanistically relevant AS changes. The links to the described analyses are available in Supplementary Table S1.

### The case study of mouse models for cardiac diseases

To demonstrate the utility of DIGGER 2.0 for mouse data analysis, we examined the RNA-seq data of cardiac disease mouse models from [6] and [7].

Acute myocardial infarction and subsequent ischemic reperfusion injury leads to complex transcriptomic changes in the heart tissue [6]. We used DIGGER 2.0 to study the functional impact of changes in AS on this cardiac condition. Without pDDIs, Whippet (|deltaPSI| > 0.1, *P*-value ≤.05) identified three Reactome pathways rewired by changes in AS between disease and healthy conditions. All three are related to cardiovascular disease and endothelial cells: “leukocyte transendothelial migration,” “RAC1 GTPase cycle,” and “RHOA GTPase cycle.” Endothelial cells can be severely disturbed in cardiac disease [53], and chronic stimulation of RHO or RAC signaling is involved in inflammatory diseases, hypertension, or decompensated heart failure [54]. Among genes where changes in AS rewire interactions with these pathways, we found RAC1 (Rac Family Small GTPase 1), which is involved in cardiac hypertrophy and heart failure onset [54].

The analysis with rMATS (|deltaPSI| > 0.05, *P*-value ≤ .05) resulted in one significantly enriched Reactome pathway: “Phase 0—rapid depolarisation.” AS changes in SCN5A and CACNA1C, which encode the subunits of the cardiac sodium and calcium channels, respectively, rewire interactions with this pathway. Mutations in both genes were shown to have a connection to the Brugada syndrome, a genetic heart disorder [55, 56].

The experimental mouse interactome is sparser than the human one. Thus, network enrichment analysis of mouse data particularly benefits from integrating pDDIs. After adding high confidence level pDDIs, we detected >300 enriched pathways, and many of them are involved in signal transduction and the immune system (Supplementary Fig. S1). The top-scoring pathway in the analysis with pDDIs is the “MAPK signaling” pathway. We also found the “Hypertrophic cardiomyopathy” and “Diabetic cardiomyopathy” pathways. Changes in AS of ATP5C1, DCLK2, and MAP3K4 rewire interactions with a “Hypertrophic cardiomyopathy” pathway, and of CACNA1C, TPM3, and ITGB1 rewire interactions with the “Diabetic cardiomyopathy” pathway. Among genes where AS affected the domain structure, we also found CDK8 (cyclin-dependent kinase 8) and SRC (SRC proto-oncogene, non-receptor tyrosine kinase), which are associated with cardiovascular disease [57] and cardiac remodeling [58], respectively.

We compared the results of the splicing-aware enrichment analysis by DIGGER 2.0 with the results from the differential gene expression analysis. We detected 3177 differentially expressed genes (Supplementary Table S2). The top 10 enriched Reactome pathways include the pathways involved in respiration and the process in mitochondria. The only cardiac-related pathway is “Cardiac conduction” (*P*-adjusted = .003). Thus, the splicing-aware analysis supplements the gene expression level analysis, providing more potential candidates for studying cardiac disease mechanisms.

Next, we analyzed the dataset from [7]. In this study, the authors performed transverse aortic constriction in mice to induce pressure overload for 2, 4, and 8 weeks. rMATS analysis (|deltaPSI| > 0.05, *P*-value ≤.01) detected 20 enriched Reactome pathways, many of which are relevant to the heart disease development (e.g. “Cardiac conduction”) or ion exchange (e.g. “Ion homeostasis,” “Platelet calcium homeostasis,” “Voltage gated potassium channels,” and “Ion transport by P-type ATPases”), which is important in maintaining a normal heart rhythm. Disruptions in the genes involved in ion homeostasis lead to arrhythmias [59].

We also performed the differential gene expression analysis for comparison. However, the analysis does not yield any significant results with *P*-adjusted ≤.05 (Supplementary Table S1).

Taken together, for the mouse data analysis, DIGGER 2.0 complemented or even excelled the gene-level analysis. The links to the described analyses are provided in Supplementary Table S1.

## Discussion

Compared to the first release, DIGGER 2.0 considerably expands its functionality. In particular, DIGGER 2.0 supports mouse data analysis as an important model organism. The changes we implemented also pave the way for future additions of other model organisms. We also expanded the repertoire of DDIs by adding pDDIs. Integrating NEASE into DIGGER 2.0 allows in-depth splicing enrichment analysis without coding expertise from the list of AS events to biological hypothesis generation. It allows users to create publication-ready visualizations and easily share the analysis with collaborators for further assessment and potential experimental validation.

We demonstrated the added value of these enhancements by re-analyzing RNA-seq data from MS patients and conducting an analysis of AS in cardiac diseases in mice models. In both cases, we found new pathways and genes relevant to the pathology of these diseases.

However, the most important limitation is still the number of PPIs augmented by DDIs. While pDDIs proved beneficial, they crucially depend on the quality of the prediction. PPIDM [5], the method we used here to predict DDIs, applies an easily interpretable statistical algorithm. However, it is inherently limited by the quality of the PPIs used as input. Thus, our next step will be integrating DDI predictions from other methods employing more complex machine learning models and additional input features such as physicochemical properties, e.g. ALT-IN [60]. Especially the latest development regarding deep-learning-based structural prediction, e.g. AlphaFold 3 [61], may be helpful in identifying interactions on a residue level, which provide information about isoform-specific changes in protein interaction. Another crucial aspect currently neglected is that the interactome is not static but changes dynamically between cell types and tissues. Thus, considering PPIs in the tissue-specific context will likely further improve the quality of the results in DIGGER. The new features we present in DIGGER 2.0 considerably improve the usability and make it more widely applicable. With our continued effort to expand and maintain this unique resource, we hope to further establish DIGGER as an essential tool for researchers studying the impact of AS changes on the interactome.

## Supplementary Material

gkaf384_Supplemental_Files

## Data Availability

DIGGER 2.0 is available at https://exbio.wzw.tum.de/digger/. The code is available at https://github.com/daisybio/DIGGER (https://doi.org/10.5281/zenodo.4967536). The generated datasets used in DIGGER are available at https://exbio.wzw.tum.de/digger/download. The data for the human use case are from the project GSE138614 and the data for the mouse use cases are from GSE275835 and GSE182985.
