# Supplementary material for: DIGGER 2.0: digging into the functional impact of differential splicing on human and mouse disorders
Source: Nucleic Acids Res. 2025 May 8;53(W1):W245–52. doi: 10.1093/nar/gkaf384 (PMC12230681; doi:10.1093/nar/gkaf384)
Supplement: gkaf384_Supplemental_Files [file gkaf384_supplemental_files.zip › DIGGER2.0_supplement.pdf]

Supplementary data for

DIGGER 2.0: Digging Into the Functional Impact of Differential Splicing on Human and Mouse Disorders

Elias Albrecht [1,2,†,*], **Konstantin Pelz** [1,†,*], **Alexander Gress** [3,4], **Hieu Nguyen Trung** [1], **Olga V. Kalinina** [3,5,6], **Tim Kacprowski** [7,8], **Jan Baumbach** [2,9], **Markus List** [1,10,‡], **Olga Tsoy** [2,‡]

[1]Data Science in Systems Biology, TUM School of Life Sciences, Technical University of Munich, Maximus-von-Imhof Forum 3, 85354 Freising, Germany.
[2]Institute for Computational Systems Biology, University of Hamburg, Albert-Einstein-Ring 8-10, 22761 Hamburg, Germany
[3]Helmholtz Institute for Pharmaceutical Research Saarland (HIPS), Helmholtz Centre for Infection Research (HZI), Campus E8.1, 66123 Saarbrücken, Germany
[4]Graduate School of Computer Science, Saarland University, Campus E1.3, 66123 Saarbrücken, Germany
[5]Drug Bioinformatics, Medical Faculty, Saarland University, Gebäude 15, 66421 Homburg, Germany
[6]Center for Bioinformatics, Saarland University, Campus E2.1, 66123 Saarbrücken, Germany
[7]Division Data Science in Biomedicine, Peter L. Reichertz Institute for Medical Informatics of Technische Universität Braunschweig and Hannover Medical School, Rebenring 56 Lower Saxony, 38106 Braunschweig, Germany
[8]Braunschweig Integrated Centre of Systems Biology (BRICS), Technische Universität Braunschweig, Rebenring 56 Lower Saxony, 38106 Braunschweig, Germany
[9]Institute of Mathematics and Computer Science, University of Southern Denmark, Campusvej 55, 5230 Odense, Denmark
[10]Munich Data Science Institute (MDSI), Technical University of Munich, Walther-von-Dyck-Straße 10, 85748 Garching, Germany

*To whom correspondence should be addressed. Email: elias.albrecht@tum.de
Correspondence may also be addressed to Konstantin Pelz. Email: konstantin.pelz@tum.de
†The first two authors should be regarded as Joint First Authors.
‡The last two authors should be regarded as Joint Last Authors.

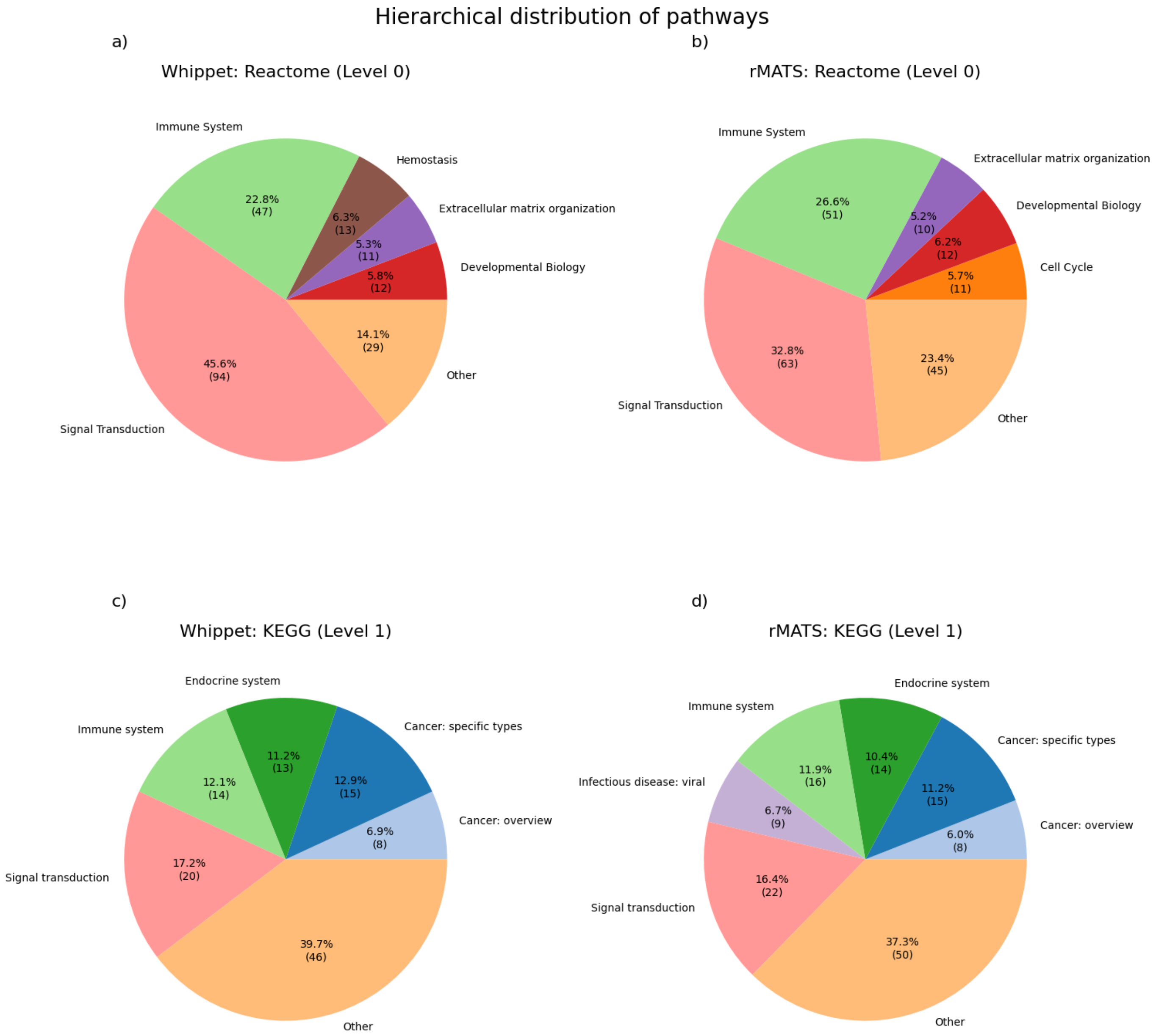

Supplementary Figure S1. Pathway analysis of the significantly enriched pathways in the mouse use case. The raw data is from Xia et al. (1). It was analyzed with rMATS and Whippet, and NEASE with standard parameters, a minimum deltaPSI of 0.1, and high-quality pDDIs were added. a) and b) are the root nodes of the Reactome pathways, and c) and d) are the first level below the root node of the KEGG pathways. This was done to make them comparable. All categories that contained 5% or less of the total amount of pathways were grouped into the "Other" category. It shows that overall methods and sources, Signal Transduction and Immune System were the most common categories.

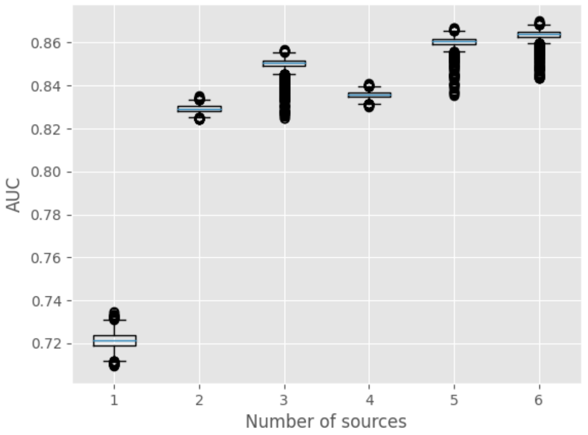

Supplementary Figure S2. Impact of the number of sources on the AUC. Each data point represents a parameter combination. Sources were selected at random, as the choice of source had a very minor impact compared to the number of sources present.

Adjusted scoring in the PPIDM method

To find optimal weights for the scoring, we modified the search strategy from an exhaustive search to a random search. Hereby, we checked 10,000 random parameter combinations and picked the one that returned the highest area under the receiver operating characteristic curve (ROC AUC) scores. This brings enormous time savings while retaining high confidence in the selected weights. For each analysis step, we chose N random sources, that support protein-protein interaction, starting from N equals to 1, and up to 6 (Fig. S2, X axis). Next, we tested 10,000 parameter combinations for each N. The general trend shows that using more sources improves the AUC while exact parameter and sources combination does not have big impact. We can also see that while some parameter combinations are outliers with a lower AUC, very few are outliers with a higher AUC.

REFERENCES

1. Xia, B., Shen, J., Zhang, H., Chen, S., Zhang, X., Song, M., and Wang, J. (2024) The alternative splicing landscape of infarcted mouse heart identifies isoform level therapeutic targets *Sci. Data,* **11**, 1154.

Table S1. Links to all NEASE analyses presented in the manuscript. The term PSI stands for 'Percentage Spliced In' and reflects the ratio between isoforms that use and splice out alternative exons. dPSI or delta PSI refers to the minimum difference of PSI values between the conditions for the event to be considered by NEASE.

Use case	preprocessing	dPSI cutoff	p-value cutoff	used predicted DDIs (confidences)	link
Multiple sclerosis	MAJIQ	0.05	0.05		https://go.tum.de/316666
Multiple sclerosis	MAJIQ	0.05	0.05	high, medium	https://go.tum.de/491923
Ischemic reperfusion	Whippet	0.1	0.05		https://go.tum.de/876894
Ischemic reperfusion	Whippet	0.1	0.05	high	https://go.tum.de/809450
Ischemic reperfusion	rMATS	0.1	0.05		https://go.tum.de/621503
Ischemic reperfusion	rMATS	0.1	0.05	high	https://go.tum.de/282553
Ischemic reperfusion	rMATS	0.05	0.01		https://go.tum.de/633534
Transverse aortic constriction	rMATS	0.05	0.01	high	https://go.tum.de/471406
